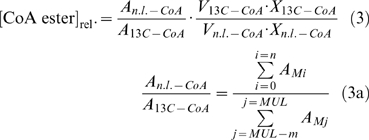# Metabolite Profiling Uncovers Plasmid-Induced Cobalt Limitation under Methylotrophic Growth Conditions

**DOI:** 10.1371/journal.pone.0007831

**Published:** 2009-11-13

**Authors:** Patrick Kiefer, Markus Buchhaupt, Philipp Christen, Björn Kaup, Jens Schrader, Julia A. Vorholt

**Affiliations:** 1 Institute of Microbiology, ETH Zürich, Zürich, Switzerland; 2 Karl-Winnacker-Institut, Dechema e.V., Biochemical Engineering, Frankfurt am Main, Germany; Max Planck Institute for Infection Biology, Germany

## Abstract

**Background:**

The introduction and maintenance of plasmids in cells is often associated with a reduction of growth rate. The reason for this growth reduction is unclear in many cases.

**Methodology/Principal Findings:**

We observed a surprisingly large reduction in growth rate of about 50% of *Methylobacterium extorquens* AM1 during methylotrophic growth in the presence of a plasmid, pCM80 expressing the *tetA* gene, relative to the wild-type. A less pronounced growth delay during growth under non-methylotrophic growth conditions was observed; this suggested an inhibition of one-carbon metabolism rather than a general growth inhibition or metabolic burden. Metabolome analyses revealed an increase in pool sizes of ethylmalonyl-CoA and methylmalonyl-CoA of more than 6- and 35-fold, respectively, relative to wild type, suggesting a strongly reduced conversion of these central intermediates, which are essential for glyoxylate regeneration in this model methylotroph. Similar results were found for *M. extorquens* AM1 pCM160 which confers kanamycin resistance. These intermediates of the ethylmalonyl-CoA pathway have in common their conversion by coenzyme B_12_-dependent mutases, which have cobalt as a central ligand. The one-carbon metabolism-related growth delay was restored by providing higher cobalt concentrations, by heterologous expression of isocitrate lyase as an alternative path for glyoxylate regeneration, or by identification and overproduction of proteins involved in cobalt import.

**Conclusions/Significance:**

This study demonstrates that the introduction of the plasmids leads to an apparent inhibition of the cobalt-dependent enzymes of the ethylmalonyl-CoA pathway. Possible explanations are presented and point to a limited cobalt concentration in the cell as a consequence of the antibiotic stress.

## Introduction

The term “metabolic burden” has been coined to describe the consequences of the presence and maintenance of plasmids in cells usually associated with a reduction of growth rate [1], [2], [3]. In addition to phenomena associated with overproduction of proteins that can strongly impact cell growth by consuming cellular resources or by interfering with host cell functions via its enzymatic activity or physical properties, different mechanisms are believed to account for plasmid-induced growth defects. Alterations in the physiological state of host cells transformed with plasmids, relative to the unmodified cells, are often ascribed to additional demands for ATP and precursors for DNA synthesis. This “metabolic burden” is supported by the observation that plasmid size correlates with a decrease in growth [4], [5], [6] and by a correlation between plasmid copy number and decreased growth [7], [8]. Although this is a generally accepted explanation provided by many authors, stoichiometric analyses showed that if no plasmid-encoded gene is expressed at high levels, even propagation of very high copy number vectors does not make much difference in global energy metabolism [9].

Diaz Ricci and Hernández [10] presented evidence for an alteration of the cellular regulatory status as a primary cause of plasmid-related growth defects. This might be the result of enzyme up-regulation leading to futile cycles or other energetically inefficient pathways [11]. In a transcriptome comparison, candidates for such up-regulated factors responsible for the “metabolic burden” were found to be heat-shock proteins, many of which are proteases or chaperones [12]. This study presents evidence for a carbon flux diversion from the glycolytic pathway to the pentose phosphate pathway as a consequence of plasmid maintenance.

The antibiotic resistance proteins encoded on many plasmids may also contribute to decreased growth rates by disturbing cellular functions. One example of toxic effects of an antibiotic resistance protein is *Serratia marcescens* lactamase *bla*_SME-1_, whose signal sequence seems to be involved in growth rate reduction of *E. coli* strains heterologously expressing this protein [13]. Especially in case of the tetracycline resistance protein TetA, which when expressed in *E. coli* was found to result in decreased growth rates by several authors. Moyed and Bertrand [14] recognized that constitutive expression of TetA from multicopy plasmids strongly affects growth and speculated about the protein's interference with the function of the cytoplasmic membrane. Another report also described the negative effect of *tetA* expression on *E. coli* fitness even in the absence of antibiotic [15].

Gaining more insight into the molecular mechanisms underlying these phenomena is of great interest for different research areas such as the spread of antibiotic resistance among pathogenic microorganisms, plasmid stability in biotechnological applications, and genetic engineering strategies. Straightforward methods such as the “omics” technologies can be used to identify cellular changes leading to growth defects of plasmid-carrying cells. While transcriptomics and proteomics provide information on gene expression and proteins, metabolomics can give a more direct view into the physiology of the cell as a consequence of the end products of gene expression and - because of the high degree of connectivity in the metabolic network - yield integrative information [16].

Methylotrophic bacteria utilize reduced carbon substrates containing no carbon-carbon bonds as their sole source of carbon and energy [17]. *Methylobacterium extorquens* AM1 is a model methylotrophic bacterium that is able to grow in the presence of methanol and methylamine. The genome of this Alphaproteobacterium was determined [18], and a number of “omics” technologies have been established and applied, helping to further expand our knowledge of methylotrophic metabolism [19], [20], [21], [22], [23]. *M. extorquens* AM1 and other methylotrophs are of interest for biotechnological applications using methanol as an alternative non-food carbon source for conversion into value-added compounds [24]. In this study, we used metabolome analysis as a means to elucidate the mechanism of the pronounced growth retardation of the model strain *M. extorquens* AM1 as a consequence of the presence of two plasmids, pCM80 and pCM160 [25], during growth in the presence of methanol as the sole carbon and energy source. Our results point to a strong reduction of ethylmalonyl-CoA mutase activity leading to inhibition of the ethylmalonyl-CoA pathway, which is necessary for glyoxylate regeneration in *M. extorquens* AM1. We provide evidence for a reduced intracellular cobalt level in plasmid-containing cells; this is most probably responsible for the decreased activity of the cobalt-dependent enzymes of the ethylmalonyl-CoA pathway.

## Results and Discussion

### Growth Characterization of *M. extorquens* in the Absence and Presence of pCM80

*M. extorquens* AM1 and *M. extorquens* AM1 with pCM80 [25], which contains in its backbone a gene conferring tetracycline resistance for maintenance and selection of the plasmid, were cultivated in a bioreactor with either succinate or methanol as the sole carbon source. In the presence of succinate, *M. extorquens* AM1 pCM80 had a growth rate μ of 0.161±0.001 h^−1^, which is only slightly below the rate observed for *M. extorquens* AM1 in absence of the plasmid and antibiotic (0.168±0.008 h^−1^). In contrast, when both strains were grown in the presence of methanol as the sole carbon source, the growth rate of *M. extorquens* AM1 pCM80 was 0.070±0.005 h^−1^ and thus was roughly half the rate of *M. extorquens* AM1 (0.158±0.003 h^−1^). Thus under methylotrophic growth conditions rather than under non-methylotrophic growth conditions, the presence of the plasmid pCM80 significantly delayed growth in this experimental system.

### Metabolite Profiling

**Figure 1:**
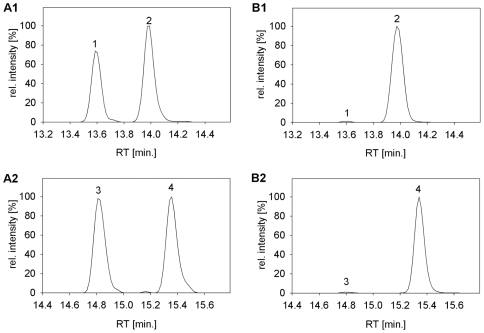
Metabolite profiling of CoA esters involved in the ethylmalonyl-CoA pathway (EMCP). Ion chromatogram sections of CoA esters extracted from *M. extorquens* AM1 (A1, A2) and *M. extorquens* AM1 pCM80 in presence of tetracycline (B1, B2). 1: succinyl-CoA; 2: methylmalonyl-CoA; 3: methylsuccinyl-CoA; 4: ethylmalonyl-CoA.

In addition to catabolic oxidation of methanol to carbon dioxide [26], [27], *M. extorquens* AM1 uses specific pathways for one-carbon (C_1_) compound assimilation that are essential for growth in the presence of methanol: the serine cycle [28], [29] in combination with the recently uncovered ethylmalonyl-CoA pathway (EMCP) [30], [31]. The serine cycle allows incorporation of C_1_ units by forming serine from glycine and methylene-tetrahydrofolate, and regeneration of the glycine precursor glyoxylate is achieved via the ethylmalonyl-CoA pathway. In order to investigate whether the decrease in growth observed as a consequence of pCM80 under methylotrophic growth conditions is linked to decreased metabolic activity of these C_1_-linked pathways, we performed targeted metabolite profiling of the respective central metabolites by ^13^C isotope dilution [32] using liquid chromatography high resolution mass spectrometry (LC-HRMS). When comparing pool sizes determined for *M. extorquens* AM1 to those found for *M. extorquens* AM1 pCM80 in the presence of tetracycline, slight changes in pool sizes of serine cycle intermediates up to a factor of about 2 were observed (Table 1). Metabolite profiling of the EMCP intermediates by LC-HRMS [31] revealed much higher relative changes for certain intermediates when comparing *M. extorquens* AM1 and *M. extorquens* AM1 pCM80 (Table 2); an increase in concentration of ethylmalonyl-CoA greater than 6-fold for *M. extorquens* AM1 pCM80 was found, and there was also a 35-fold increase of the methylmalonyl-CoA pool. Both metabolites have in common their conversion by specific mutases [33], [34] (i.e., ethylmalonyl-CoA mutase catalyzing the conversion of ethylmalonyl-CoA to methylsuccinyl-CoA, methylmalonyl-CoA mutase converting methylmalonyl-CoA to succinyl-CoA). These cofactor B_12_-dependent enzymes play a key role in glyoxylate regeneration in *M. extorquens* AM1 [31] and are essential for growth on methanol whereas this pathway is dispensable for growth on succinate [26]. Comparison of chromatographic peaks of ethylmalonyl-CoA and methylsuccinyl-CoA, as well as methylmalonyl and succinyl-CoA, obtained from LC-HRMS analysis (Fig. 1) revealed that the product and educt of the mutase catalyzed reactions are very similar in *M. extorquens* AM1. However, in *M. extorquens* AM1 pCM80, an important accumulation of the substrates of the reactions in the direction of the net flux of the EMCP was observed (Fig. 1). These results suggest a bottleneck within the EMCP specifically at the mutase reactions. However, since we cannot determine the specific S/R configuration of the substrates of the two mutase reactions using the metabolome analyses applied, it is also conceivable that the mutase-preceding step, ethylmalonyl-CoA/methylmalonyl-CoA epimerase, is affected. This protein has also been shown to be stimulated by Co^2+^ [33], [35], [36]. As a consequence, down-regulation of gene expression or enzyme inhibition might occur and lead to the observed accumulation of pool sizes and ultimately reduced growth.

**Table 1:** Metabolite profiling of *M. extorquens* AM1 and *M. extorquens* AM1 pCM80 during growth on methanol.

	Metabolite		AM1		AM1 pCM80	
Serine cycle	2-/3-Phosphoglycerate	a	1.00	±0.11	0.41	±0.03
		b	1.42	±0.13	0.47	±0.04
	Phosphoenolpyruvate	a	0.51	±0.03	0.48	±0.08
		b	0.74	±0.05	0.34	±0.09
	Pyruvate	a	0.56	±0.08	1.47	±017
		b	0.64	±0.04	1.12	±0.17
	Malate	a	2.33	±0.10	0.89	±0.14
		b	2.41	±0.13	0.58	±0.04
	Serine	a	1.39	±0.11	1.08	±0.47

Average and standard deviation of metabolite pool sizes are given in μmol/g_Cell dry weight_. Values shown for *M. extorquens* AM1 were taken from a previous study [21]. a and b represent two biological replicates.

**Table 2:** Comparison of ethylmalonyl-CoA pathway intermediates extracted from *M. extorquens* AM1 pCM80 in presence of tetracycline and from *M. extorquens* AM1 during growth on methanol.

Compound	Fold change A		Fold change B	
Succinyl-CoA	1.09	±0.08	1.82	±0.16
Methylmalonyl-CoA	35.53	±3.50	1.43	±0.15
Mesaconyl-CoA	1.45	±0.13	1.45	±0.11
Ethylmalonyl-CoA	6.44	±1.01	0.54	±0.10
Methylsuccinyl-CoA	0.49	±0.11	1.78	±0.40
Acetyl-CoA	0.48	±0.04	0.96	±0.09
3-Hydroxybutyryl-CoA	0.43	±0.03	0.93	±0.05
Propionyl-CoA	3.57	±0.26	0.81	±0.06
Crotonyl-CoA	0.78	±0.18	1.50	±0.47

Values are the fold changes of CoA ester pool sizes of strain AM1 pCM80 relative to AM1. A: when applying default cobalt concentration; B: when applying 10 times the default cobalt concentration.

### Cobalt Dependency of Glyoxylate Regeneration *In Vivo*

The pCM80 plasmid harbors a gene providing resistance to tetracycline via active efflux of tetracycline out of the cell [37]. Tetracycline efflux requires divalent cations for export, forming a chelate complex with the dication with the highest affinity to Co^2+^ for all investigated ions [38]. It seems reasonable to assume that mutases and/or epimerase and tetracycline compete for free cobalt. The strong efflux of tetracycline may thus cause a drop in the intracellular cobalt concentrations below a threshold level required for sufficient activity of the cobalt-dependent enzymes of the EMCP, which would result in accumulation of ethylmalonyl-CoA and methylsuccinyl-CoA, reduced carbon flux through EMCP, and ultimately reduced growth in the presence of methanol.

**Figure 2:**
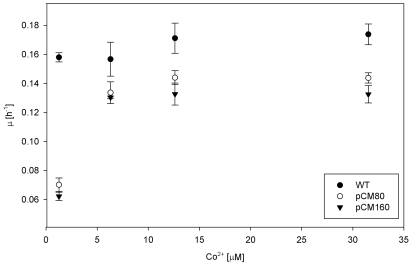
Influence of cobalt concentration on growth. Growth rates of *M. extorquens* AM1 strains observed during exponential growth on minimal medium with different cobalt concentrations. Values were determined for the wild-type strain (WT), strain AM1 with pCM80 plasmid coding for tetracycline resistance, and strain AM1 pCM160 plasmid coding for kanamycin resistance. Average values and standard deviations were calculated from three biological replicates carried out in shake flasks.

In order to evaluate whether cobalt limited growth of *M. extorquens* AM1 pCM80, the strain as well as wild type were cultivated in shake flasks on minimal medium with methanol containing different cobalt concentrations, and growth rates were determined (Fig. 2). When the cobalt concentration was 10 times increased (12.6 µM), the measured growth rate of *M. extorquens* pCM80 doubled (0.144±0.005 h^−1^) corresponding to 84% of the growth rate of *M. extorquens* AM1. For the latter only a slight increase of less than 10% was observed with increasing cobalt concentration (0.171±0.010 h^−1^). Similar results were observed when cobalt concentration was increased 25 times and 100 times, though in the latter case a slight decrease of growth rates was observed for both strains (Fig. 2). The results show that the strong growth limitation of *M. extorquens* AM1 pCM80 in the presence of tetracycline during growth on minimal medium with methanol as the sole carbon source could be partially compensated by increasing the cobalt concentration in the medium. In a second step, we cultivated *M. extorquens* AM1 pCM80 in a bioreactor in the presence of tetracycline on minimal medium with methanol containing 10 times increased cobalt concentration. Similar to the results obtained from cultivations in shake flasks, two biological replicates revealed growth rates of 0.13 h^−1^ and 0.14 h^−1^. EMCP intermediates were extracted during exponential growth. Table 2, column B shows the fold changes of EMCP intermediates relative to the wild-type strain; all CoA ester pools were comparable to those observed for wild-type. Moreover, the accumulation of methylmalonyl-CoA and ethylmalonyl-CoA was no longer detectable, indicating that addition of cobalt overcomes the metabolic inhibition.

### Introduction of Isocitrate Lyase

Heterologous expression of isocitrate lyase can partially restore glyoxylate regeneration in case the authentic system is blocked [39]. For further evidence that glyoxylate regeneration limits growth of *M. extorquens* AM1 pCM80, growth of *M. extorquens* pCM80*icl* was determined. *M. extorquens* pCM80*icl* was cultivated on methanol with the same cultivation conditions as *M. extorquens* AM1 pCM80. Two biological replicates resulted in growth rates of 0.11 h^−1^ and 0.13 h^−1^, respectively. Compared to *M. extorquens* AM1, growth rates were partially restored when isocitrate lyase was introduced, a result that is in line with the assumption that cobalt limitation is the primary cause of growth delay via blocking the EMCP.

### Characterization of *M. extorquens* in the Presence of pCM160

In the case that the mechanism of tetracycline resistance was the reason *M. extorquens* became low on intracellular cobalt in the presence of pCM80 and tetracycline, the presence of pCM160, which carries a gene for kanamycin resistance instead of the tetracycline resistance gene, should not affect growth in the same way pCM80 does. The kanamycin resistance gene encodes a nucleotidyltransferase that catalyzes the transfer of a nucleoside monophosphate group to the 4′-hydroxyl group of the aminoglycoside kanamycin to inactivate the antibiotic [40]. To our knowledge there is no indication for an involvement of cobalt ions in the mechanism of action of this enzyme. To test the hypothesis of a less severe growth phenotype due to the presence of pCM160, *M. extorquens* AM1 pCM160 was cultivated on minimal medium with either methanol or succinate, in presence of the antibiotic. Three biological replicates yielded a growth rate of 0.142±0.01 h^−1^ on succinate, a rate that was about 15% below that of the wild-type strain (0.168±0.01 h^−1^). Surprisingly, on methanol the growth rate was 0.062±0.003 h^−1^, which was similar to that observed for *M. extorquens* pCM80. When performing cultivation on minimal medium with methanol as the sole carbon source at different cobalt concentrations, the growth rate increased significantly with increasing cobalt concentration in the medium, as observed for strain pCM80 (Fig. 2). When cobalt concentration was increased 10 times, the growth rate more than doubled (0.133±0.008 h^−1^), and similar growth rates were observed for higher cobalt concentrations, corresponding to 78% of the wild-type growth rate. Thus, poor methanol growth of the pCM160 strain could also be compensated by increasing the cobalt concentration in the medium (Fig. 2).

For further comparison, *M. extorquens* AM1 pCM160 was cultivated in shake flasks in the presence of kanamycin on minimal medium with natural labeled methanol containing the default cobalt concentration and on minimal medium with 99% ^13^C methanol containing a 10 times increased cobalt concentration. Cells were grown to a similar OD, and samples from both cultures were taken in parallel, mixed, and quenched at the same time. CoA esters were extracted from sample mixtures and subsequently analyzed by LC-HRMS. To evaluate reproducibility, the experiment was repeated applying ^13^C-labeled methanol to minimal medium with the default cobalt concentration. In addition, the same experiment was carried out with *M. extorquens* AM1 pCM80 in the presence of tetracycline as a control. Growth rates on minimal medium with default cobalt concentrations were 0.072 h^−1^ and 0.065 h^−1^ for *M. extorquens* AM1 pCM80, and 0.068 h^−1^ and 0.066 h^−1^ for *M. extorquens* AM1 pCM160. With a 10 times increased cobalt concentration, *M. extorquens* AM1 pCM80 yielded growth rates of 0.12 h^−1^ and 0.13 h^−1^, and *M. extorquens* AM1 pCM160 growth rates were 0.12 h^−1^ and 0.13 h^−1^. Thus, cobalt concentration had the same influence on growth on methanol for both strains. Table 3 shows the fold change of EMCP metabolites when increasing to ten times the cobalt concentration in the medium. Not only did both strains show very similar decreases of ethylmalonyl-CoA and methylmalonyl-CoA, but also, all measured EMCP intermediates underwent similar changes for both strains when cobalt concentration was increased. The results for *M. extorquens* pCM160 together with those obtained for *M. extorquens* pCM80 suggest that perturbation of intracellular cobalt concentration might be due to a more general response mechanism of antibiotic stress of *M. extorquens* AM1 rather than to the specific mechanism of tetracycline resistance, although a combination of the two effects is possible.

**Table 3:** Growth of *M. extorquens* AM1 pCM80 on methanol in the presence of tetracycline and of *M. extorquens* AM1 pCM160 in the presence of kanamycin.

Metabolite		Fold change pCM80			Fold change pCM160	
Succinyl-CoA	a	1.39	±0.25		1.41	±0.01
	b	1.32	±0.02		1.74	±0.08
Methylmalonyl-CoA	a	0.06	±0.01		0.04	±<0.01
	b	0.06	±<0.01		0.04	±<0.01
Mesaconyl-CoA	a	0.90	±0.06		0.73	±<0.01
	b	0.84	±0.03		0.84	±0.05
Ethylmalonyl-CoA	a	0.16	±<0.01		0.13	±<0.01
	b	0.13	±<0.01		0.15	±0.01
Methylsuccinyl-CoA	a	1.68	±0.38		1.77	±0.06
	b	1.45	±0.06		1.75	±0.11
Crotonyl-CoA	a	2.01	±0.34		1.73	±0.29
	b	2.41	±0.16		2.39	±0.20
Acetyl-CoA	a	1.77	±0.06		1.43	±<0.01
	b	1.86	±0.03		1.85	±0.06
3-Hydroxybutyryl-CoA	a	2.54	±0.03		2.23	±0.02
	b	2.22	±0.03		3.17	±0.12
Propionyl-CoA	a	0.29	±0.04		0.22	±0.01
	b	0.29	±0.01		0.26	±0.01
Crotonyl-CoA	a	2.01	±0.34		1.73	±0.29
	b	2.41	±0.16		2.39	±0.20

Values are the fold changes of metabolite pools sizes of the ethylmalonyl-CoA pathway when 10 times the default cobalt concentration was present in medium. a and b represent two biological replicates.

### Overproduction of a Putative TonB-Dependent Cobalt Transporter

As propagation of either pCM80 or pCM160 caused very similar effects on growth rates and EMCP metabolite ratios, we tried to find another explanation for the plasmid-induced intracellular cobalt depletion. The exposure of cells to antibiotics can lead to the activation of a stress response, which includes changes in the expression of outer membrane proteins [41]. To identify potential candidates for *M. extorquens* cobalt transporters, which could be down-regulated due to exposure to tetracycline or kanamycin in cells containing pCM80 or pCM160, we searched the genome for factors involved in cobalt uptake. In an *in silico* comparative genomic analysis to identify prokaryotic nickel and cobalt uptake transporters, a specific group of TonB-dependent receptors was presented based on conserved regulatory motifs and conserved colocalization in nickel or cobalt transport system operons [42]. Experimental evidence for a nickel transport function across the outer membrane by a protein from this group has been provided [43]; this clearly extends the substrate spectrum of TonB-energized transport, previously thought to be restricted to iron complexes and cobalamin compounds [44]. By performing a BLAST search on the *M. extorquens* genome with the hypothetical TonB-dependent cobalt transporter Daro_1684 from *Dechloromonas aromatica* [42], [44] we identified two TonB-dependent receptors with high similarity to the *D. aromatica* protein: META2_0097, which has been designated a putative TonB-dependent receptor for iron transport by homology, and META1_1076, a TonB-dependent transporter homologous to numerous other uncharacterized bacterial outer membrane transporters. As META1_1076 most probably forms an operon with META1_1077, the latter encoding a putative periplasmic homolog of the cobalt chelatase CbiK, META1_1076 seemed to be a promising candidate for an outer membrane cobalt transporter.

Therefore we transformed an overexpression construct of this operon, pCM80-Mext META1_1076/META1_1077, into *M. extorquens* AM1 and determined the growth rate μ in methanol medium containing the default cobalt concentration and tetracycline. We found a growth rate of 0.122±0.003 h^−1^, corresponding to roughly 80% of the growth rate of *M. extorquens* AM1 in methanol medium. Overexpression of this TonB-dependent transport system thus suppressed the growth defect caused by limiting intracellular cobalt concentration in plasmid-bearing cells, suggesting that the observed growth defect phenomenon might be caused by antibiotic stress-mediated repression of this outer membrane cobalt transport system. Notably, a very recent study identified the same transporter system for cobalt uptake by an evolutionary experiment with *M. extorquens* AM1 on metal deficient growth media utilizing methanol as carbon source [45]. It was shown that increased expression of this transporter in the mutant strain yielded increased growth rates under cobalt limitations compared to the ancestor strain. It is currently unclear whether the aforementioned putative TonB-dependent receptor META2_0097, which is annotated as an iron transporter, might also contribute to cobalt transport across the outer membrane in *M. extorquens* AM1. *M. extorquens* AM1 also contains a second putative periplasmic cobalt chelatase (META1_0967), as well as an operon encoding two factors known to constitute a cytoplasmic membrane cobalt transport system (CbtA (META1_1332) and CbtB (META1_1333)). Overexpression of the CbtA/B operon using a pCM80 construct did not result in an increase in growth rate compared to *M. extorquens* AM1 pCM80 (data not shown), suggesting that transport across the inner membrane might not be limiting under the antibiotic stress conditions.

### Conclusions

It is well known that groups of microorganisms have a relatively high demand for certain trace elements that represent metal sites of key metabolic enzymes. Schönheit et al. discovered that at low concentrations of nickel (less than 100 nM), cobalt (less than 10 nM), and molybdenum (less than 10 nM), the quantity of *Methanothermobacter marburgensis* cells formed was roughly proportional to the amount of transition metal added to the medium when cells were grown on H_2_ and CO_2_ [46]. Later the enzymes, such as methyl-CoM reductase (Ni), hydrogenases (Ni), acetyl CoA synthase/CO dehydrogenase (Ni), methyl transferases (Co), and formylmethanofuran dehydrogenase (Mo), were identified [47]. When methanogens are grown in the presence of methanol, cobalt was found to become the primary limiting trace element [48]. Another example is the alkyl *tert*-butyl ether-degrading Betaproteobacteria, which was shown to exhibit an elevated nutritional demand for cobalt due to the presence of a cobalamin-dependent mutase during growth on substrates possessing the *tert-*butyl moiety [49].

Here we demonstrated that metabolic profiling represents a useful method to observe bottlenecks in metabolism as a consequence of shortages of trace elements. This is of interest for the purposes of elucidation of novel pathways with yet uncharacterized enzymes and of engineering strategies for biotechnological applications. From the results it is obvious that cobalt is a critical trace element for methylotrophic growth of *Methylobacterium extorquens*, not only in the presence of antibiotics but also for the wild type in dependence upon the medium and concentrations employed. One may imagine that the high cobalamin requirement during growth in the presence of methanol makes *Methylobacterium* a good expression host for cobalamin-dependent enzymes. Our study also shows that the utilization of plasmids for strain improvement strategies might sometimes be critical; although the introduction of *icl* to the pCM80 plasmid results in a higher growth rate of *M. extorquens* compared to the empty plasmid, *M. extorquens* AM1 pCM80*icl* is still growing slower than the plasmid-free *M. extorquens* AM1 since the isocitrate lyase only partially compensates for inhibited activity of EMCP pathway introduced by pCM80 plasmid under the default experimental conditions.

## Materials and Methods

### Chemicals and Medium Composition

^13^C methanol (99%) was purchased from Cambridge Isotope Laboratories. All other chemicals were purchased from Sigma-Aldrich. Acetonitrile, acetic acid, formic acid, and ammonium hydroxide used for HPLC solvents were of LC-MS degree. *M. extorquens* AM1 strains were grown on minimal medium as described previously [31], except that the default concentration of CoCl_2_⋅6H_2_O (0.3 mg L^−1^) was modified for a number of cultivation experiments (see Results). In the presence of the pCM80 plasmid and the pCM160 plasmid, 10 mg L^−1^ of tetracycline and 50 mg L^−1^ of kanamycin were added to the medium, respectively. Both plasmids are versatile broad-host-range vectors introduced for use in Gram-negative bacteria and in methylotrophs in particular. They are based on the pCM62 and pCM66 plasmids that were shown to be maintained in *M. extorquens* AM1 with equal efficiency [25].

### Plasmid Construction

DNA cloning was performed using standard methods [50]. To construct plasmid pCM80-MetA1_1076/MetA1_1077, the operon containing ORFs MetA1_1076 and MetA1_1077 was amplified by PCR using primers TCGATCTAGAATGTCGCCGCTTCTGACGC and AGCTGAGCTCTCAGTTGGACTTCGGGTCGGTG with chromosomal DNA of *M. extorquens* AM1 as template. After digestion of the PCR product and plasmid pCM80 [25] with *Xba*I and *Sac*I, fragments were ligated. The *E. coli* isocitrate lyase gene was amplified from genomic *E. coli* DNA by PCR using primers ATATAAGCTTAACTATGGAGCATCTGCACA and TATATCTAGATTAGAACTGCGATTCTTCAG. After digestion of the PCR product and plasmid pCM80 with XbaI and HindIII, fragments were ligated to construct pCM80*icl*.

### Cultivation Conditions

All pre-cultures were carried out in baffled shake flasks at 28°C and 110 rpm. Main cultures in shake flasks were carried out under the same conditions. Cultures were inoculated to an initial OD_600_ between 0.05 and 0.1. Main cultures in a bioreactor were carried out in a 500 mL bioreactor (Infors-HT, Bottmingen, Switzerland) with a 400 mL working volume at 28°C and at 1000 rpm. The pH was maintained at 7.0 by addition of 1 M ammonium hydroxide. Initial OD_600_ was about 0.3. Three independent cultivation experiments were carried out for shake flask experiments and two in case of bioreactor experiments.

### Sampling, Quenching, and Extraction of Metabolites for Quantification

For the purpose of metabolic profiling experiments, cells were harvested during mid-exponential growth at 2<OD_600_<3. In case of small polar compounds (amino acids, organic acids, and small phosphorylated metabolites), a cell amount corresponding to 0.6 mg cell dry weight was sampled by fast filtration [51]. The latter included a wash step to remove metabolites eventually present in the culture liquid phase. Metabolites were extracted over 8 min with boiling water [21]. For metabolite quantification the isotope dilution method was applied [32]. To this end, uniformly ^13^C-labeled cell extract from *M. extorquens* AM1 cells grown on methanol was added to the sample as internal standard when starting metabolite extraction. In the case of CoA esters, sampling and quenching of samples was performed as described [31]. Similarly, for small polar metabolites, uniformly ^13^C-labeled cell extract from *M. extorquens* AM1 grown on methanol was added simultaneously with the sample to the quenching solution. For extraction, the sample was incubated for 15 min on ice. During incubation, samples were mixed for 10 s every 5 min. After addition of 20 mL of ice-cold deionized water, samples were chilled with liquid nitrogen. Frozen samples were stored at −20°C until freeze drying. When a LC-HRMS protocol with an improved sample desalting method was applied (see below), freeze dried samples were dissolved in 80 µl of ice-cold, 25 mM ammonium formate buffer (pH 3.5, 2% methanol), the suspension was centrifuged (14,000 g, 3 min, 0°C), and the supernatant was used for LC-HRMS analysis. Otherwise, samples were prepared as previously described [31].

### LC-HRMS Analysis

All analyses were done using a Rheos 2200 HPLC instrument (Flux Instruments, Switzerland) with an LTQ Orbitrap mass spectrometer (Thermo Fisher Scientific), equipped with an electrospray ionization probe. Small polar compounds were analyzed as described previously [21]. CoA esters were analyzed as described [31] or by applying a modified protocol with significantly improved desalting of the samples. For both protocols solvent A was 50 mM formic acid adjusted to pH 8.1 with ammonium hydroxide, and solvent B was methanol. The flow rate was 220 µL min^-1^, and the injection volume was 10 µL. In the case of the alternative protocol, a C_18_ analytical column (Gemini 50×2.0 mm, particle size 3 µm, Phenomenex) used for desalting was connected via a 6-port valve to a second C18 analytical column (Gemini 100×2.0 mm, particle size 3 µm). To desalt the sample, it was injected on the short column, which was connected to the waste. During desalting the flow of the second column was provided by a second HPLC pump. After 5 min the two columns were connected in line. The gradient of B was as follows: 0 min, 0%; 5 min 0%; 15 min, 23%; 25 min, 80%; 27 min, 80%; 27.5 min, 0%; 32 min, 0%. MS analysis was performed in the negative or in the positive FTMS mode at a resolution of 30,000. For the positive FTMS mode, sheath gas flow rate was 40, aux gas flow rate was 30, tube lens was 80 V, capillary voltage was 30 V, and ion spray was 4.7 kV. For the negative FTMS mode, gas flow rates were identical, tube lens was −90 V, capillary voltage was −4 V, and ion spray current was −4.7 kV.

### Data Analysis

Growth rates were determined from exponential fits of growth curves using the Matlab curve fitting tool. The goodness of fit criterion was an adjusted R^2^ value higher than 0.99. For metabolite quantification from cultivations in bioreactors the isotope dilution method developed by [32] was applied by adding U-^13^C labeled cell extract (internal standard) to the sample prior to metabolite extraction, and concentrations were calculated as follows:
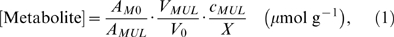
where A_MUL_ is the area of the uniformly ^13^C-labeled mass peak, A_M0_ is the area of the monoisotopic mass peak, V_0_ is the sample volume, V_MUL_ is the volume of the U-^13^C labeled cell extract, c_MUL_ is the known concentration of the U-^13^C labeled metabolite of the internal standard, and X is the biomass concentration. When the absolute concentration of a metabolite in the internal standard was unknown, relative differences in pool sizes were calculated from normalized peak areas as follows:
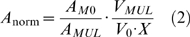



For a set of cultivations in shake flasks CoA ester pool sizes of strains AM1 containing the plasmids pCM80 and pCM160 were compared to those of the wild type growing under default conditions applying the approach developed by Mashego *et al.* [52]. To this end *M. extorquens* AM1 wild type was cultivated on default medium containing 99% U-13C whereas *M. extorquens* AM1 pCM80 and *M. extorquens* AM1 pCM160 were grown on natural labeled methanol. Defined amounts of ^13^C-labeled cells were quenched and extracted together with defined amounts of natural labeled extracts. Mass isotopomer distributions of CoA esters were determined by LC-MS. Since cultivations were carried out in shake flasks natural labeled CO_2_ from air was present. CoA esters formed on ^13^C methanol in shake flasks showed an increased fraction of partially ^13^C-labeled CoA esters though the mass isotopomer distributions of CoA esters formed on natural labeled and ^13^C-labeled methanol were completely separated. The observed increase of ^12^C carbon can be explained by operation of the ethylmalonyl-CoA pathway during growth on methanol incorporating a significant fraction of CO_2_ into biomass [31]. Therefore, to determine the relative difference between natural labeled and ^13^C-labeled CoA ester pool sizes [CoA ester]_rel._ all mass peaks M_0_, M_1_, …, M_n_ measured from natural labeled CoA esters (n.l.-CoA) and all mass isotopomers M_MUL-m_, …, M_MUL-1_, M_MUL_ from CoA esters formed on ^13^C methanol (13C-CoA) were taken into account, and relative pool sizes of CoA esters were calculated as follows: